# Maternal Factors in Pregnancy and Ethnicity Influence Childhood Adiposity, Cardiac Structure, and Function

**DOI:** 10.3389/fped.2022.900404

**Published:** 2022-07-19

**Authors:** Sophia Khan, Andrew Whatmore, Reena Perchard, Aysha Khan, Avni Vyas, Jaspal Dua, J. Kennedy Cruickshank, Peter Clayton

**Affiliations:** ^1^Division of Developmental Biology and Medicine, Faculty of Biology, Medicine and Health, Manchester Academic Health Science Centre, University of Manchester, Manchester, United Kingdom; ^2^North West, North Wales and Isle of Man Adult Congenital Heart Disease Network, Liverpool Heart and Chest Hospital NHS Foundation Trust, Liverpool, United Kingdom; ^3^School of Life-Course and Nutritional Sciences, King’s College, St Thomas’ and Guy’s Hospitals, King’s Health Partners, London, United Kingdom

**Keywords:** cardiovascular risk, maternal factors during pregnancy, childhood anthropometrics, echocardiography, ethnicity

## Abstract

**Importance:**

The links between maternal and offspring adiposity and metabolic status are well established. There is much less evidence for the impact of these relationships combined with ethnic background on cardiac structure and function in childhood.

**Objective:**

To test the hypothesis that ethnicity, maternal adiposity and glycemic status, and child adiposity affect cardiac structure and function.

**Design:**

A prospective cohort study.

**Setting:**

A single-center mother-child cohort study. The cohort is a subset of the international multi-center Hyperglycemia and Adverse Pregnancy Outcomes (HAPO) study.

**Participants:**

This study included 101 healthy pre-pubertal British-born children [56 White Europeans (WEs) and 45 South Asians (SAs)] with a median age of 9.1 years, range 6.0–12.2 years, at the time of the investigation.

**Main Outcomes and Measures:**

Anthropometric and echocardiographic measurements were made on the cohort. Maternal pregnancy and birth data were available. Relationships between maternal parameters (BMI and glucose status), child adiposity, and echo measures were assessed.

**Results:**

Despite no ethnic difference in BMI SDS at a median age of 9.1 years, SA children exhibited higher levels of body fat than WE children (whole body, right arm, and truncal fat all *p* < 0.001). SA children also exhibited greater changes in weight and height SDS but not BMI SDS from birth than WE children. As expected, maternal BMI correlated with child BMI (*r* = 0.28; *p* = 0.006), and body fat measures (e.g., whole body fat *r* = 0.25; *p* = 0.03). Maternal fasting glucose levels were associated with child body fat measures (*r* = 0.22–0.28; *p* = 0.02–0.05). Left ventricular (LV) indices were not different between SA and WE children, but E/A and E′/A′ (measures of diastolic function) were lower in SA when compared with WE children. LV indices correlated positively to BMI SDS and body fat markers only in SA children. Maternal fasting and 2-h glucose were negatively correlated with E′/A′ in SA children (*r* = −0.53, *p* = 0.015, and *r* = −0.49, *p* = 0.023, respectively) but not in WE children.

**Conclusion and Relevance:**

SA and WE children exhibit differences in adiposity and diastolic function at a median age of 9.1 years. Novel relationships between maternal glycemia, child adiposity, and cardiac structure and function, present only in SA children, were identified.

## Introduction

Cardiovascular disease (CVD) accounts for 25% of adult deaths in the United Kingdom and over 43,000 a year are “premature,” occurring in those younger than 75 years (1–3). It is well recognized that the pathophysiology of standard and novel risk factors, including maternal factors, begins in early life.

Intrauterine growth restriction and lower birth weight increases later CVD risk, (4) while fetal over-nutrition, other metabolic aspects of the intrauterine environment, and early growth patterns are also linked to adult CVD and other non-communicable diseases (5). One example is the association of rapid “catch-up growth” with increased CVD risk in later life (6–8).

Increased CVD risk in adults of South Asian (SA) origin when compared with Europeans (9) is partly due to the higher prevalence of certain CV risk factors, such as dyslipidemia, diabetes, and hypertension (10). Other factors such as a genetic predisposition and adverse body fat patterning are also likely to contribute, as well as unknown factors (11). A recent prospective UK biobank data study confirmed that the SA CVD risk is not explained by disparities in risk factors alone (12).

The Manchester Heart and Growth Study (MHGS), a subset of the Hyperglycemia and Adverse Pregnancy Outcome (HAPO) study (13), reflects the ethnic diversity of the local population. We used this cohort to investigate the impact of maternal BMI and glycemic status in pregnancy on cardiac structure and function in SA when compared with European-origin children, and how this was affected by childhood adiposity. We tested the hypothesis that ethnicity, maternal adiposity and glycemic status, and child adiposity affect cardiac structure and function.

## Participants and Methods

This study included 101 healthy British-born children (56 WEs, 45 SAs), of which 59 had echocardiograms. The median age of the children was 9.1 years, range 6.0–12.2 years, and all were pre-pubertal (Tanner stage 1) at the time of the investigation. The mean gestational age of the children in the cohort was 39.6 weeks (range 30.0–42.1). There were no significant differences between the gestational age of the WE and SA children in the cohort.

As part of the HAPO Study (13), mothers had undergone an oral glucose tolerance test (OGTT), at around 28 weeks of gestation (range 24–32 weeks) using a 75 g glucose load, with height, weight, and blood pressure (BP) measured.

The children in the Manchester cohort of the HAPO study were followed up as part of the Manchester Heart and Growth Study (MHGS) in which data were collected at birth, and at subsequent time points, further anthropometric and cardiovascular data were collected (14, 15). The protocol was expanded to include bio-impedance measures and echocardiography.

All studies were conducted with Local Research Ethics committee (LREC) approval. All mothers gave written informed consent for their children, and children assented where required by the LREC.

All equipment was calibrated to ensure accuracy. Standing height was measured to the nearest 0.1 cm, using a wall-mounted stadiometer and weight was measured to the nearest 0.1 kilogram using digital scales. Skin fold thicknesses were measured to the nearest 0.1 cm using metal calipers at the following sites: subscapular, triceps, and supra-iliac. The means of triplicate measurements were used in the analysis.

Bio-impedance was measured using TANITA™ body composition analyzer scales (Tanita, United Kingdom). Whole-body, trunk, and limb bio-impedance (Ω) and percentage fat were measured. Body fat reference curves for bio-impedance data in children were available to facilitate interpretation (16). BMIs were also calculated.

Children rested for 3 min prior to measuring systolic and diastolic BP (in mmHg) by a Dinamap Pro 400 monitor (GE Healthcare, United Kingdom) with appropriate age-validated cuffs. Measurements were repeated three times, with a minute’s rest between measures. The mean of the triplicate readings was used for analysis.

Echocardiography (Phillips iu-22 echo, Phillips, United Kingdom) included two-dimensional (2D) echo, M-mode, Doppler studies, and Tissue Doppler (TD), with images stored for subsequent off-line speckle tracking analysis. From the parasternal long-axis view, using 2D echo and M-Mode, a full left ventricular (LV) study was carried out. Interventricular septum (IVS) and LV posterior wall (LVPW) thickness and LV internal diameter at end-diastole (LVIDd) and end-systole (LVIDs) were measured from the M-Mode image. LV mass (LVM) measurement was also derived (17) and normalized to body surface area (BSA).

The E/A ratio, a marker of diastolic function, was assessed using pulse wave Doppler (PWD) through the mitral valve (Supplementary Figure 1) to measure early (E) and late (A) inflow velocities to calculate E/A. An impairment of relaxation, as seen in early diastolic dysfunction, will cause a reduction in the E/A ratio.

Tissue Doppler imaging (TDI) is volume independent and provides information similar to cardiac MRI while being readily available and not requiring sedation. TDI was used to assess diastolic function by measuring the peak velocities at the mitral valve annulus. As with conventional PWD, TDI gives an E wave and an A wave, and these are referred to as E’ and A’. As seen for E/A ratios, reduced diastolic function (where relaxation is impaired) is associated with a lower E′/A′ ratio.

Statistical analysis was carried out using SPSS (Version 23). Independent samples *T*-tests were used to assess differences between groups with *p* < 0.05 being taken as significant. Non-parametric (Spearman) correlations and generalized linear models (GLM) were used to examine relationships between maternal and childhood anthropometric and CV data.

With regard to power, for the key measure of E/A with our sample size, we had a 94% chance of detecting a difference between groups at a type 1 error rate (α) of 0.05.

## Results

### Ethnic Differences in Children’s Anthropometry

SA newborns had lower birth weights, lengths, and BMIs than their WE counterparts in keeping with previous reports (14, 18). By a median age of 9.1 years of age, there was no significant difference in weight, height, or BMI SD scores between SA and WE children consistent with catch-up growth in SA children (Table 1). In the whole cohort, the weight, height, and BMI SD scores were all significantly higher at a median age of 9.1 years when compared with those at birth. The change in weight and height SDS but not BMI SDS was significantly higher in SA when compared with WE children (Table 1).

**TABLE 1:** Anthropometric data from birth to the median age of 9.1 years.

	All	WE	SA
Weight SDS at birth	−0.33 (1.37)	**0.08 (1.19)**	−0.84 (1.41)*
Height SDS at birth	−0.27 (1.15)	**0.06 (1.18)**	−0.66 (1.00)*
BMI SDS at birth	−0.25 (1.33)	**0.08 1.09)**	−**0.65 (1.49)**^#^
Weight SDS age 9y	0.78 (1.09)	0.77 (0.92)	0.80 (1.27)
Height SDS age 9y	0.53 (0.97)	0.42 (0.88)	0.66 (1.05)
BMI SDS age 9y	0.71 (1.22)	0.79 (0.97)	0.63 (1.45)
Change in weight SDS birth to 9y	1.09 (1.56)	**0.59 (1.48)**	**1.69 (1.46)** ^‡^
Change in height SDS birth to 9y	0.81 (1.36)	**0.29 (1.39)**	**1.42 (1.04)** ^‡^
Change in BMI SDS birth to 9y	0.91 (1.65)	0.62 (1.42)	1.26 (1.85)

Weight SDS, height SDS, and BMI SDS measurements were compared in WE (N = 44 at birth and 53 at 9y) and SA children (N = 36 and 46). Values are mean (SD), with significant differences between ethnic groups highlighted and represented by: ^#^p ≤ 0.05, *p ≤ 0.01, and ^‡^p ≤ 0.001. Bold indicates significant results.

Despite comparable weights and BMI SDS at a median age of 9.1 years, all measures of body fat (total, right arm, and truncal) were significantly higher in SA when compared with WE children, as was supra-iliac skin fold thickness (SISFT), a measure of truncal fat (Table 2).

**TABLE 2:** Body fat and skin fold thickness (SFT) measures at a median of 9.1 years.

	WE	SA
**Body fat%**		
Whole body	23 (5)	28 (8)*
Right arm	32 (5)	37 (7)*
Truncal	17 (5)	22 (7)*
**Skin folds mm**		
Supra-iliac SFT	10.3 (6.0)	15.3 (8.7)*
Triceps SFT	13.6 (4.7)	15.3 (6.3)
Subscapular SFT	10 (5.8)	12.2 (7.0)

Body fat (%) was measured in WE (n = 41) and SA (n = 35) children by bio-impedance, while skin fold thickness (mm) was measured in WE (51) and SA (n = 46) children using calipers. Values are mean (SD) with significant differences between ethnic groups represented by: *p ≤ 0.01.

### Ethnic Differences in Maternal Data

Data from the 28-week OGTT for 51 WE and 45 SA mothers showed no significant differences in BMI (28 ± 4.1 vs. 28 ± 5.2 kg/m^2^, *p* = 0.8), maternal glucose concentrations (either fasting; WE 4.6 ± 0.4 vs. SA 4.7 ± 0.5 mmol/L, *p* = 0.2, or at 2-h; 6.2 ± 1.3 vs. 6.5 ± 1.8 mmol/L, *p* = 0.3), systolic BP (107 ± 11 vs. 104 ± 11 mmHg, *p* = 0.2), and diastolic BP (70 ± 8 vs. 70 ± 9 mmHg, *p* = 0.5).

### Maternal Data and Offspring’s Anthropometry

Maternal BMI was positively correlated with children’s BMI at a median age of 9.1 years (*r* = 0.28, *p* = 0.006) with similar significant correlations between maternal BMI and children’s whole body fat (*r* = 0.25, *p* = 0.03), truncal fat (*r* = 0.25, *p* = 0.04) and all three SFTs (*r* = 0.24–0.27, *p* = 0.01–0.02) (Supplementary Table 1), as found in the full HAPO follow-up study (19). When analyzed by ethnicity, correlations between maternal BMI and SISFT (*r* = 0.35, *p* = 0.02) in WE children and between maternal BMI and truncal fat (*r* = 0.35, *p* = 0.05) and triceps SFT (*r* = 0.3, *p* = 0.05) in SA children were significant.

In the whole cohort, maternal fasting glucose was significantly correlated with offspring whole body fat (*r* = 0.28, *p* = 0.02), right arm fat (*r* = 0.23, *p* = 0.05), truncal fat (*r* = 0.23, *p* = 0.05), and SISFT (*r* = 0.22, *p* = 0.04) (Supplementary Table 1). GLM with the children’s whole body fat or truncal fat as the dependent variable showed SA ethnicity and female gender were the most significant covariates (Supplementary Table 2).

### Ethnic Differences in Children’s Cardiac Structure and Function

59 children (31 WE, 28 SA) had echocardiography. LV mass indexed for body surface area (BSA), IVSd or LVPWd was not significantly different between WE and SA children (Supplementary Table 3).

E/A was measured by PWD in 55 children (28 WEs, 27 SAs) and E′/A′ by tissue Doppler in 44 children (22 WEs, 22 SAs). Diastolic function was significantly lower in SA children when compared with WE children when measured by either E/A (WE: 2.1 ± 0.4 vs. SA: 1.7 ± 0.4, *p* = 0.002) or E′/A′ (WE: 3.2 ± 0.6 vs. SA: 2.7 ± 0.9, *p* = 0.03) (Table 3). This was a result of higher A waves being observed in SA children.

**TABLE 3:** Ethnic difference in Children’s diastolic function.

	WE	SA
E (cm/s)	98.1 (12.7)	96.8 (14.2)
A (cm/s)	48.4 (9.1)	56.2 (10.5)*
E/A	2.1 (0.4)	1.7 (0.4)*
E’ (cm/s)	18.6 (2.6)	18.4 (3.4)
A’ (cm/s)	5.6 (1.2)	6.9 (2.8)^#^
E’/A’	3.2 (0.6)	2.7 (0.9)^#^

Echocardiography was used to measure early (E), late (A) waves, the E/A ratio, and E′, A′ and the E′/A′ ratio as markers of diastolic function in WE (n = 28) and SA (n = 27) children. Values are mean (SD) with significant differences between ethnic groups represented by: ^#^p ≤ 0.05 and *p ≤ 0.01.

### Children’s Adiposity and Cardiac Structure and Function

In WE children, there were no significant relationships between cardiac structure (as measured by LVM indexed to either height or BSA, IVSd, or LVPWd) and any indices of body mass or fat (Table 4). In contrast, in SA children, BMI SDS, whole-body fat, arm fat, and truncal fat were all significantly correlated to LVPWd (all ≤ 0.01) and to IVSd (BMI SDS *p* < 0.01, body fat measures *p* < 0.04) (Table 4).

**TABLE 4:** Correlations (Spearman) between child’s body composition and cardiac measures by ethnic group (WE *n* = 28 and SA *n* = 27).

		WE				SA		
				
	BMI SDS	Whole-body fat	Arm fat	Truncal fat	BMI SDS	Whole-body fat	Arm fat	Truncal fat
IVSd	0.21	0.16	0.01	0.16	0.52*	**0.45** ^#^	**0.52** ^#^	**0.50** ^#^
LVPWd	−0.15	−0.29	−0.32	−0.25	0.52*	0.60*	0.61*	0.62*
LVIDd	0.19	0.12	−0.09	0.09	**0.41** ^#^	0.32	0.21	0.34
LVM/BSA	−0.01	−0.10	−0.23	−0.16	0.04	0.08	0.02	0.13

Significant correlations are represented by: ^#^p ≤ 0.05, *p ≤ 0.01.

*IVSd, Interventricular septum thickness at end-diastole; LVPWd, Left ventricular posterior wall thickness at end-diastole; LVIDd, Left ventricular internal diameter at end-diastole; LVM/BSA, Left ventricular mass corrected for body surface area. Bold indicates significant results.*

In the whole cohort, E/A was significantly negatively correlated with whole-body (−0.32; *p* = 0.03), arm (−0.35; *p* = 0.02), and truncal fat (−0.35; *p* = 0.02). When analyzed by ethnicity, these correlations did not reach significance.

Across the whole cohort and in WE alone, changes in weight, height, and BMI SDS from birth to a median age of 9.1 years showed no significant correlations to cardiac structure or function at a median age 9.1 years. However, in SA children, change in weight SDS correlated positively with IVSs (*r* = 0.57; *p* = 0.007), while change in BMI SDS correlated positively with IVSs (*r* = 0.59; *p* = 0.005), LVPWd (*r* = 0.49; *p* = 0.02), and LVPWs (*r* = 0.54; *p* = 0.01).

### Maternal Factors and Cardiac Structure and Function

No significant associations were identified between maternal BMI and LV indices of diastolic function. Fasting but not 2-h maternal glucose negatively correlated with diastolic function (E′/A′) in the whole cohort (*r* = −0.45, *p* = 0.003). This correlation was significant in SA children (*r* = −0.53, *p* = 0.015) but not in WE children (*r* = −0.02, *p* = 0.92). In SA children, E′/A′ was also related to maternal 2-h glucose in the OGTT (*r* = −0.49, *p* = 0.023).

The relationship between maternal glucose and diastolic function (E/A and E′/A′) was further investigated using a generalized linear model with E/A or E′/A′ as the dependent variable and ethnicity, gender, maternal glucose, and BMI as co-variates. Maternal fasting glucose was negatively related to E′/A′ (β = −0.93, *p* < 0.001) with a significant ethnic effect (*p* = 0.02), while ethnicity (*p* = 0.003) was the only significant variable relating to E/A (Table 5).

**TABLE 5:** Generalized Linear Modeling with Child’s E′/A′ or E/A as dependent variables and ethnicity (reference group SA), gender (reference group Male), maternal fasting glucose, and maternal BMI as covariates.

Marker	β	Std. Error	95% CI			
			
			Lower	Upper	Chi-square	*P*-value
**E’/A’**						
Ethnicity	**0.48**	0.21	0.08	0.89	5.58	**0.02**
Gender	0.06	0.21	−0.36	0.47	0.07	0.80
Maternal fasting glucose	−**0.93**	0.28	−1.48	−0.37	10.7	**<0.001**
Maternal BMI	0.02	0.02	−0.03	0.06	0.64	0.42
**E/A**						
Ethnicity	**0.32**	0.11	0.11	0.53	8.65	**0.003**
Gender	0.02	0.11	−0.19	0.23	0.04	0.85
Maternal fasting glucose	0.03	0.14	−0.24	0.30	0.06	0.81
Maternal BMI	0.001	0.01	−0.02	0.03	0.01	0.94

*Significant covariates are highlighted in bold with the p-value shown.*

## Discussion

The relationship between maternal metabolic status in pregnancy and its impact on infant and childhood growth and metabolism has long been of interest in keeping with the “Developmental Origins of Health and Disease” hypothesis. The HAPO study demonstrated that maternal glycemic status well below that associated with diabetes was associated with increased birth weight and increased cord blood C-peptide levels. The HAPO follow-up study, returning to the same mother-child pairs, has demonstrated that maternal BMI and glucose levels have a long-lasting impact on the child’s adiposity and glycemic status (19–22).

We have used our Manchester cohort from the HAPO study, which was predominantly composed of British-born WE and SA children, to examine relationships found in the HAPO studies and additional questions related to cardiac structure and function in a healthy population brought up in the same city. This was due to the higher rate of CVD among SA adults in the United Kingdom. In our first study, we demonstrated that contrary to our hypothesis, SA infants had significantly lower markers of inflammation when compared with WE infants over the first 2 years of life, with early growth being unrelated to C-reactive protein or interleukin-6 levels (15). Thus, inflammatory markers did not reflect increased cardio-metabolic risk at this age. However, when assessed up to 3 years of age, we did find that SA children had lower adiponectin but similar HDL-cholesterol and insulin levels when compared with WE children (23).

We have also shown a lower birth weight in SA infants with ethnic and gender differences in growth and adiposity present in early infancy. Differences included truncal fat preservation in SA girls from birth and SA boys showing a striking early increase in length and weight, associated with increased subscapular fat when compared with WE boys (Tables 1, 2). In addition, those with the highest weight change over the first 3 months have the highest systolic BP at 12 months (14).

In the current study undertaken at a median of 9.1 years of age, SA children, despite comparable BMI SDS, had greater SISFT (a measure of truncal adiposity) and a higher proportion of body fat in all areas (Table 2). Both ethnic groups demonstrated an increase in BMI and weight SDS from birth to a median of 9.1 years, with the increases in height and weight SDS being significantly higher in SA children (Table 1). These findings support the hypothesis that early growth patterns and “catch-up growth” in SA children result in the development of potentially adverse body fat patterns, which may increase later CVD risk.

BMI that is unsuitable when describing adiposity in SA children confirms previous studies highlighting the inadequacy of BMI in describing overweight and obesity in SA children (24–27). Increased body fat in SA adults when compared with WE adults with the same BMI has, in part, been attributed to differences in muscularity (28). Our findings demonstrate that these ethnic differences in fat distribution originate in early childhood.

Maternal BMI has been linked to childhood overweight/obesity (29, 30); this is supported by results presented here with associations in both WE and SA children. In addition, in our whole cohort, maternal fasting glucose was related to adiposity measures. Recent reports from the HAPO follow-up study in which the children are now aged 10–14 years also identify maternal glucose levels, independent of BMI, being associated with increased childhood overweight/obesity and with a child’s glucose and insulin sensitivity (20, 22, 31, 32). It is suggested that *in utero* exposure to high free fatty acids and maternal glucose leads to permanent changes in metabolism, neuroendocrine functions, appetite control, and an increased later obesity risk. The epidemiological implications of these theories of obesity programming are important, as female offspring can carry forward this programming to subsequent generations which could lead to an exponential rise in obesity (5).

Our study has now focused on childhood cardiac structure and function to test the hypothesis that maternal metabolic impact and child adiposity would influence heart development and that this would show the ethnic disparity. Animal studies have shown associations between maternal obesity and cardiac hypertrophy and dysfunction due to the effects of higher insulin levels (33). In addition, intrauterine and early life factors including maternal smoking and low birth weight have been associated with children’s left ventricular structure (34).

Despite a large amount of literature reporting associations between childhood obesity, systemic hypertension, and diabetes with cardiac structure and function, there is a paucity of research investigating these associations in multi-ethnic cohorts of healthy children (35–39). Large United Kingdom studies such as those of Barker and the ALSPAC cohort predominately include those of WE origin (40, 41). Other mixed ethnicity United Kingdom childhood cohort studies do exist including the “Born in Bradford/Growing up in Bradford” study where 45% of the participants are of SA origin and the CHASE study (participants of SA, black African-Caribbean, and WE origin) (26, 42). These studies have not included echo measures to assess cardiac structure and function. The IndEcho study has recently been established with an aim to investigate the relationship between size at birth, childhood growth, and CVD risk markers in young adult life with myocardial structure and function in midlife in South Asians (43). Recruitment for this study is from two previous Indian birth cohorts and is investigating individuals living in India, not the United Kingdom; results are not yet available.

In contrast to the above studies, we are reporting findings in two healthy British-born cohorts, one of European descent and the other South Asian, brought up in the same city. In this population, we did not find any major differences in left ventricle measurements between WE and SA children (Supplementary Table 3). However, in the SA children but not the WE children, adiposity measures correlated with various left ventricular dimensions, suggesting that adiposity affects the hearts of SA children differently from WE children (Table 4). A recent report found no evidence of fetal cardiac programming related to gestational diabetes mellitus or maternal pre-pregnancy obesity on left ventricle function in Finnish children at the age of 6 years, in keeping with our findings in WE children (44).

We did find that diastolic function, as measured by E/A and E′/A′, was lower in SA children than in WE children (Table 3). Although in the whole cohort, diastolic function was negatively related to adiposity, there was only a weak correlation of BMI SDS to E′/A′ in WE children but not SA children. This inverse relationship between body fat and CV function has recently been reported in overweight/obese children when compared with lean controls (45). Studies in obese children have also reported that despite preserved systolic cardiac function, there is sub-clinical diastolic dysfunction. This is due to impaired myocardial relaxation as indicated by reduced mitral inflow velocities (38, 39, 46). There are also reports that children with increased CV risk due to underlying chronic conditions such as renal disease or diabetes have sub-clinical diastolic dysfunction which may be associated with other subtle changes in CV status (37, 46, 47).

In addition, we demonstrated that higher maternal glucose (both fasting and 2-h glucose in OGTT) in pregnancy was associated with lower diastolic function in SA children but not in WE children: this may indicate that the intrauterine environment could adversely affect offspring’s later cardiac health and that SA children may be more susceptible.

These ethnic differences in diastolic function may suggest that the increased CV risk seen in SA adults begins with sub-clinical changes in cardiac function in childhood and that monitoring diastolic function in childhood may allow detection prior to overt clinical disease and thereby allow early intervention.

Potential limitations of this study include the risk of bias, confounding factors, and chance associations. Longitudinal cohort studies are at risk of attrition bias: in this cohort, loss to follow-up did not differ by ethnicity or socio-economic group. In addition, the growth characteristics of participants in this study did not differ from those not enrolled (data not shown). An important limitation of this study is that we do not have data on the participant’s lifestyle, diet, and other behaviors, such as exercise, which may impact the relationships between maternal and child characteristics and cardiovascular parameters. In addition, our cohort is relatively small and cannot be used to prove causality without further validation in independent cohorts.

In conclusion, this study extends our knowledge of the relationships between maternal adiposity and glycemic status in pregnancy and outcomes in their children in the pre-adolescent years in the following ways: (1) there are distinct differences in adiposity markers, early growth patterns, and cardiac function between the United Kingdom born healthy WE and SA children with the latter having more truncal fat and lower diastolic function, (2) maternal BMI is related to childhood adiposity, an effect that is seen predominantly in girls, while maternal glycemic status in pregnancy affects diastolic function in SA children. These findings demonstrate that the impact of both the *in utero* environment and ethnic background on a child’s adiposity markers and cardiac structure and function can be detected in mid-childhood, and these relationships should be considered in public health programs to improve child and later-life health.

## Data Availability Statement

The raw data supporting the conclusions of this article will be made available by the authors, without undue reservation.

## Ethics Statement

The studies involving human participants were reviewed and approved by the Local Research and Ethics Committee. Written informed consent to participate in this study was provided by the participants’ legal guardian/next of kin.

## Author Contributions

SK, AW, and PC designed the study, analyzed the data, and wrote the manuscript while SK, AV, and AK collected the data. All authors reviewed and approved the submitted manuscript.

## Conflict of Interest

The authors declare that the research was conducted in the absence of any commercial or financial relationships that could be construed as a potential conflict of interest.

## Publisher’s Note

All claims expressed in this article are solely those of the authors and do not necessarily represent those of their affiliated organizations, or those of the publisher, the editors and the reviewers. Any product that may be evaluated in this article, or claim that may be made by its manufacturer, is not guaranteed or endorsed by the publisher.

## Abbreviations

A: Peak velocity of late mitral in-flow
A’: Peak velocity of late diastolic mitral annular motion
BSA: Body surface area
CVD: Cardiovascular disease
E: Peak velocity of early diastolic mitral in-flow
E’: Peak velocity of early diastolic mitral annular motion
E/A: Ratio of peak velocity of early diastolic mitral in-flow to peak velocity of late mitral in-flow
E’/A’: Ratio of peak velocity of early diastolic mitral annular motion to peak velocity of late mitral annular motion
HAPO: Hyperglycemia and Adverse Pregnancy Outcomes
IVS, Interventricular septum: IVSs at end-systole: IVSd at end-diastole
LV: Left ventricular
LVID, Left ventricular internal diameter: LVIDs at end-systole: LVIDd at end-diastole
LVPW, Left ventricular posterior wall thickness: LVPWs at end-systole: LVPWd at end-diastole
MHGS: Manchester Heart and Growth Study
SA: South Asian
SISFT: Supra-iliac skin fold thickness
TDI: Tissue Doppler imaging
WE: White European.
