# Supplementary material for: Maternal Factors in Pregnancy and Ethnicity Influence Childhood Adiposity, Cardiac Structure, and Function
Source: Front Pediatr. 2022 Jul 19;10:900404. doi: 10.3389/fped.2022.900404 (PMC9343669; doi:10.3389/fped.2022.900404)
Supplement: Supplementary file 1 [file Data_Sheet_1.pdf]

Supplementary Figures and Tables

Suppl. Figure 1: Image of a 2D echo 4-Chamber view, with the pulse wave cursor through the mitral valve. At the bottom of the image is the Pulse Wave Doppler trace of the mitral inflow velocities with the E and A waves labelled.

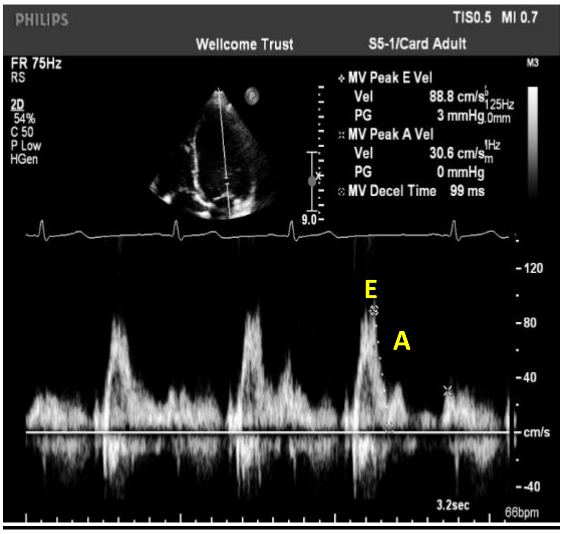

Suppl. Table 1: Correlations of maternal data with body fat at median 9 years. Maternal glucose (fasting and 2-hour post OGTT) and BMI were measured at 28 weeks and correlated with child's body composition taken at median 9 years of age. Correlation coefficients (r) are shown with with p values ($^{\#}$ p≤ 0.05 and * p≤ 0.01).

**Maternal data ↓**	**Child data →**	**BMI**	**Body fat**	**Arm fat**	**Truncal fat**	**Supra iliac SFT**	**Triceps SFT**	**Subscapular SFT**
**Fasting glucose**		0.07	0.28$^{\#}$	0.23$^{\#}$	0.23$^{\#}$	0.22$^{\#}$	0.14	0.14
**2hr glucose**		0.11	0.23$^{\#}$	0.26$^{\#}$	0.20	0.15	0.17	0.07
**BMI**		0.28*	0.25$^{\#}$	0.19	0.25$^{\#}$	0.24$^{\#}$	0.26*	0.27*

Suppl. Table 2: Generalised Linear Modelling with Child's Whole Body Fat or Truncal Fat (%) as dependent variable and ethnicity (reference group SA), gender (reference group male), maternal fasting glucose and maternal BMI as covariates. Significant values (in bold) are represented by: # p≤ 0.05, * p≤ 0.01 and ‡ p≤ 0.001.

A: Whole cohort:

Marker	β	Std. Error	95% CI		Chi-square
			Lower	Upper	
**Whole Body Fat:**					
Ethnicity	-5.3	1.3	-7.9	-2.8	**16.6** ‡
Gender	4.6	1.3	2	7.2	**11.7** ‡
Maternal Fasting Glucose	0.9	1.7	-2.5	4.2	0.3
Maternal BMI	0.3	0.2	0.03	0.7	**4.6** #
**Truncal Fat:**					
Ethnicity	-5.1	1.2	-7.5	-2.8	**18.4** ‡
Gender	3.4	1.2	1	5.8	**7.7** *
Maternal Fasting Glucose	1.0	1.6	-2.0	4.1	0.4
Maternal BMI	0.3	0.2	-0.01	0.6	3.5

B: By gender:

Marker	β	Std. Error	95% CI		Chi-square
			Lower	Upper	
**Whole Body Fat:**					
Females					
Ethnicity	-7.2	2.1	-11.2	-3.1	**12.1** ‡
Maternal Fasting Glucose	-2.9	2.2	-7.3	1.5	1.7
Maternal BMI	1.0	0.3	0.4	1.5	**12.1** ‡
Males					
Ethnicity	-5.7	1.5	-8.6	-2.8	**14.4** ‡
Maternal Fasting Glucose	5.9	2.4	1.1	0.7	**5.7** #
Maternal BMI	-0.1	0.2	-0.4	0.3	0.2
**Truncal Fat:**					
Females					
Ethnicity	-6.6	1.9	-10.4	-2.8	**11.5** ‡
Maternal Fasting Glucose	-1.9	2.1	-6.0	2.3	0.8
Maternal BMI	0.8	0.3	0.3	1.3	**9.5 ***
Males					
Ethnicity	-5.5	1.4	-8.3	-2.8	**15.9** ‡
Maternal Fasting Glucose	4.7	2.3	0.23	9.1	**4.3** #
Maternal BMI	-0.1	0.2	-0.4	0.3	0.2

Suppl. Table 3: Comparison of left ventricular indices. Values are given as mean (SD). All p values for T-Tests are non-significant (>0.05)**.**

	WE	SA
**Left Ventricular Mass/BSA**	65.03 (14.5)	60.35 (10.52)
**IVS diastole**	7.11 (0.99)	7.04 (0.95)
**LVID diastole**	39.12 (4.37)	37.08 (3.64)
**LVPW diastole**	6.61 (1.14)	6.5 (1.2)
**IVS systole**	10.17 (1.3)	9.52 (1.36)
**LVID systole**	24.24 (3.48)	23.16 (2.85)
**LVPW systole**	10.56 (1.68)	10.19 (1.63)
